# Sweet taste receptors play roles in artificial sweetener-induced enhanced urine output in mice

**DOI:** 10.1038/s41538-023-00236-9

**Published:** 2024-01-05

**Authors:** Shuangfeng Cai, Ningning Xie, Ling Zheng, Quan Li, Siyu Zhang, Qinghua Huang, Wei Luo, Mei Wu, Yidan Wang, Yilun Du, Shao-ping Deng, Lei Cai

**Affiliations:** 1 Engineering Research Center of Molecular Medicine of Ministry of Education, Key Laboratory of Fujian Molecular Medicine, Key Laboratory of Xiamen Marine and Gene Drugs, School of Biomedical Sciences and School of Medicine, Huaqiao University, Xiamen, 361021 PR China; 2 Food Safety Key Laboratory of Zhejiang Province, School of Food Science and Biotechnology, Zhejiang Gongshang University, Hangzhou, 310018 PR China; 3 Women’s Hospital, School of Medicine, Zhejiang University, Hangzhou, 310006 PR China

**Keywords:** Feeding behaviour, Digestive signs and symptoms

## Abstract

Sweet taste receptors found in oral and extra oral tissues play important roles in the regulation of many physiological functions. Studies have shown that urine volume increases during the lifetime exposure to artificial sweeteners. However, the detailed molecular mechanism and the general effects of different artificial sweeteners exposure on urine volume remain unclear. In this study, we investigated the relationship between urinary excretion and the sweet taste receptor expression in mice after three artificial sweeteners exposure in a higher or lower concentration via animal behavioral studies, western blotting, and real-time quantitative PCR experiment in rodent model. Our results showed that high dose of acesulfame potassium and saccharin can significantly enhance the urine output and there was a positive correlation between K^+^ and urination volume. The acesulfame potassium administration assay of T1R3 knockout mice showed that artificial sweeteners may affect the urine output directly through the sweet taste signaling pathway. The expression of T1R3 encoding gene can be up-regulated specifically in bladder but not in kidney or other organs we tested. Through our study, the sweet taste receptors, distributing in many tissues as bladder, were indicated to function in the enhanced urine output. Different effects of long-term exposure to the three artificial sweeteners were shown and acesulfame potassium increased urine output even at a very low concentration.

## Introduction

Artificial sweeteners (ASs) are widely used as substitutes for sugars in the formulation of food and beverage because of their properties, such as providing an intense sweetness with no or few calories^1^. However, some studies suggest that the consumption of low-calorie ASs may be linked to weight gain^2^, increased abdominal fat deposition^3^, type 2 diabetes^4^, and cardiovascular disease^5^. The potential risks of ASs, including saccharin, cannot be overlooked.

The sensory system of mammals, with taste being one of its most essential abilities^6^, is crucial in our daily life. Studies have shown that the activation of sweet receptors on taste cells by sweet molecules initiates a common signaling pathway^7,8^. Low-calorie ASs can activate the signaling cascade associated with T1R2/T1R3 receptors and gustducin protein Gα to trigger calcium-mediated signaling pathways^9^, which are vital in many physiological processes. Consequently, disturbances in Ca^2+^ metabolism can induce many major chronic diseases, such as osteoporosis, kidney disease, obesity, heart disease, and hypertension^10^.

More and more taste signaling components have been found in a variety of non-taste organs over recent years^11^, including the pancreas, heart, brain, stomach, small intestine, testes, kidneys^12^, and the bladder^13^. It was reported that these receptors participate in physiological metabolism^13^, and in these organs, T1Rs may play a key role in detecting nutrients or performing other physiological functions. Studies suggest that the stomach receives chemosensory signals directly by macronutrients via T1R3 homodimers, and T1R3 is also involved in regulating ghrelin release after glucose intake^14,15^. Moreover, T1R3 and the Gα protein exhibited differential expression levels during various periods of mouse development^16^.

Over the past decades, a number of researchers have sought to determine the relationship between ASs and urine output. Although kidney play a vital role in producing urine, the urine output had been related to other organs function, such as the bladder contraction^17,18^. Recent evidence suggests that saccharin is slowly absorbed from the gut, but is not metabolized and rapidly eliminated in the urine via excretion by the kidneys^19^. Since saccharin is not metabolized, the Food and Drug Administration regards it as “safe”^20^. Importantly, several physiological changes have been discovered after sodium saccharin (NaSac) exposure, including increased urine volume, increased sodium concentration^21^. Recently, investigators have shown, via immunostaining, that the sweet taste receptors T1R2 and T1R3 are expressed in the human and rat bladder urothelium^22^, especially in the umbrella cells. It was also shown that the in-vitro test of ASs could probably lead to bladder contraction by altered ion concentration of urine, including K^+^, Cl^-^, and Ca^2+^, indicating a relationship between ASs and the bladder^23^. Although extensive research has been carried out on the effect of ASs, no single study exists to illuminate whether T1Rs taste receptors take part in the molecular mechanism.

The Acceptable Daily Intake (ADI) is defined as the amount of additives that can be safely consumed over a lifetime without posing a risk to health. The ADI for saccharin and sucralose is currently 5 and 15 mg/kg/day. However, the limit for acesulfame potassium (AceK) is 9 mg/kg/day^20^. In this study, a potential risk of AS usage had been observed even in the concentration of ADI, the physiological function of sweet taste receptors in non-oral organ needs to be investigated. Therefore, we aimed to investigate other risks associated with exposure to ASs at the ADI or higher dose concentration in a rodent model. Additionally, this study seeks to elucidate the underlying mechanisms of T1Rs and the effect of orally ingested ASs at different concentrations on increased urine output.

## Results

### Physiological effects of artificial sweeteners exposure

**Fig. 1:**
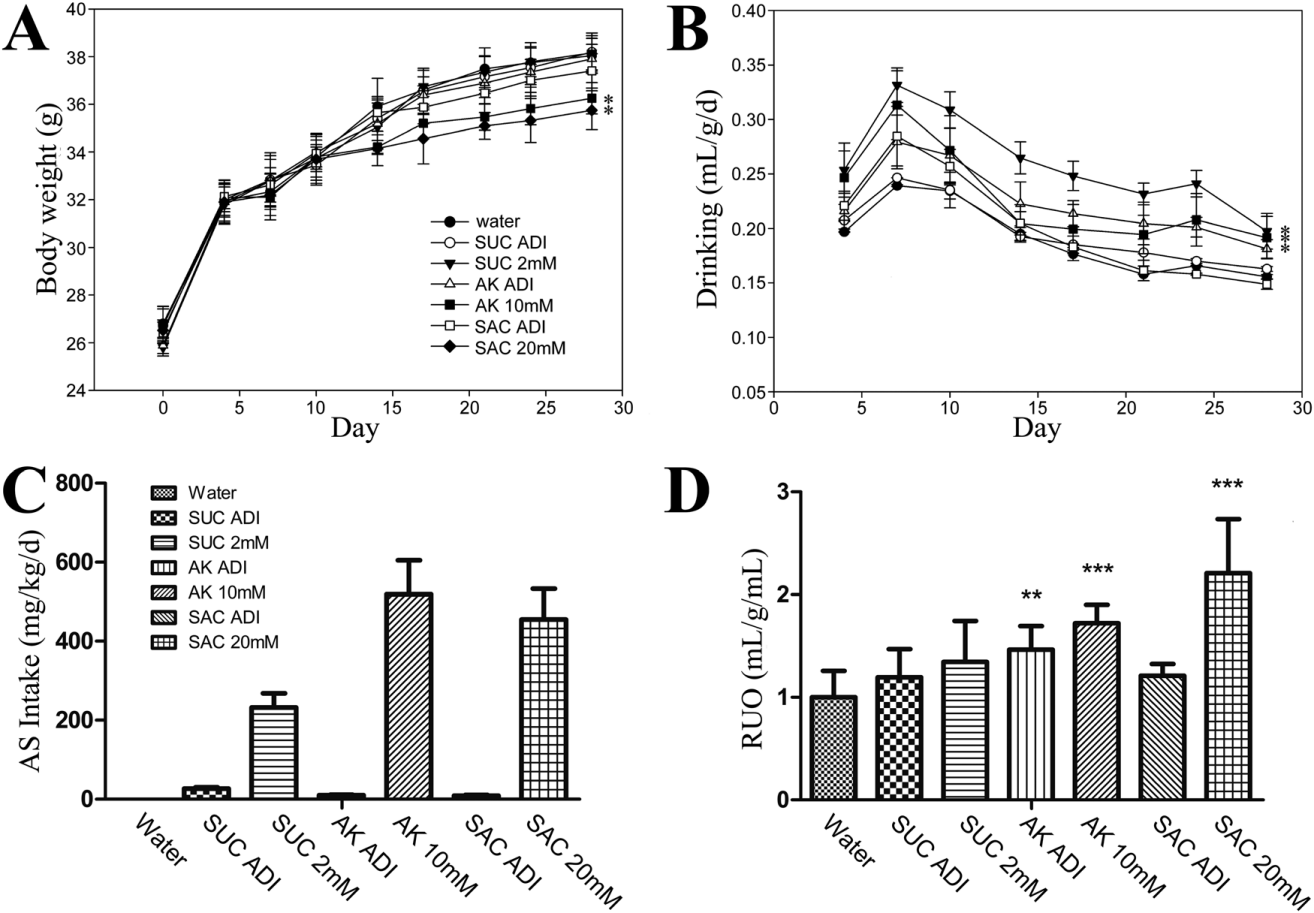
Physiological effect of mice with different concentrations of artificial sweeteners orally exposure for 4 weeks (*n* = 10). **A** body weight; (**B**) drinking; (**C**) the amount of artificial sweetener intake; (**D**) Relative urine output (RUO) in mice. Graph (**D**) shared the same legend with (**C**). SUC: sucralose; AK acesulfame potassium, SAC saccharin. The water group was used as the negative control. One-way ANOVA and Dunnett’s multiple comparison post-hoc test were performed for all results. All the error bars show the standard deviations of the means. *P < 0.05; **P < 0.01; ***P < 0.001.

A four-week ASs exposure was set to determine the physiological effect. The ADI concentration of all ASs had no effect on body weight, higher concentration AceK and saccharin had a greater effect, but not in higher sucralose group. The body weights were significantly reduced in the group of AceK 10 mM and saccharin 20 mM (Fig. 1A). For daily drinking, the high dose groups of all three sweeteners were significantly increased (Fig. 1B). Although there were some differences in daily drinking, the daily intake of ASs conformed to the experimental design, which means the intake of high concentration group is significantly higher than that of low concentration group (Fig. 1C).

**Fig. 2:**
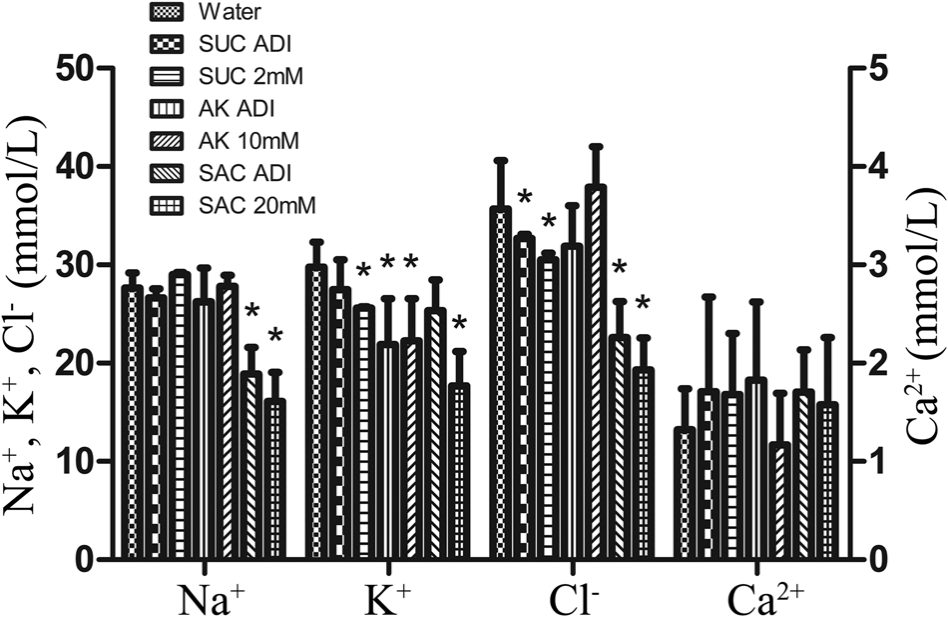
Ion concentration measurement in urine after artificial sweeteners orally exposure for 4 weeks. The concentrations of Na^+^, K^+^, Cl^-^ and Ca^2+^in urine (*n* = 10 in each group). SUC sucralose, AK acesulfame potassium, SAC saccharin. The water groups were used as the negative controls, respectively. Dunnett’s multiple comparison post-hoc test was performed for all results. Error bars show the standard deviations of the means. *P < 0.05.

Furthermore, we observed a substantial difference in the relative urine output (RUO) analysis within different groups. Compared to the water control group, the RUO were significantly increased in both ADI and 10 mM group of AceK. A dramatic increase of RUO was found in saccharin 20 mM group, but not in the ADI group. Neither ADI nor 2 mM group of sucralose showed statistically significant difference (Fig. 1D). Between the experimental groups, the impact was also apparent. The effect on the saccharin group was the greatest when compared with all other groups. The urine output in the low-dose AceK group was even higher than that in the sucralose high-dose group. The ion concentrations in urine were drastically changed after ASs exposure. For Na^+^, only saccharin group showed a significant change compared to water group. K^+^ in all the AceK group, high dose group of sucralose and saccharin were significantly reduced. No significant changes of Cl^-^ were found in AceK group, and the concentrations of Ca^2+^ were comparable among all groups (Fig. 2).

### Sweet taste receptors in bladder are regulated by artificial sweetener administration and affect urine volume

**Fig. 3:**
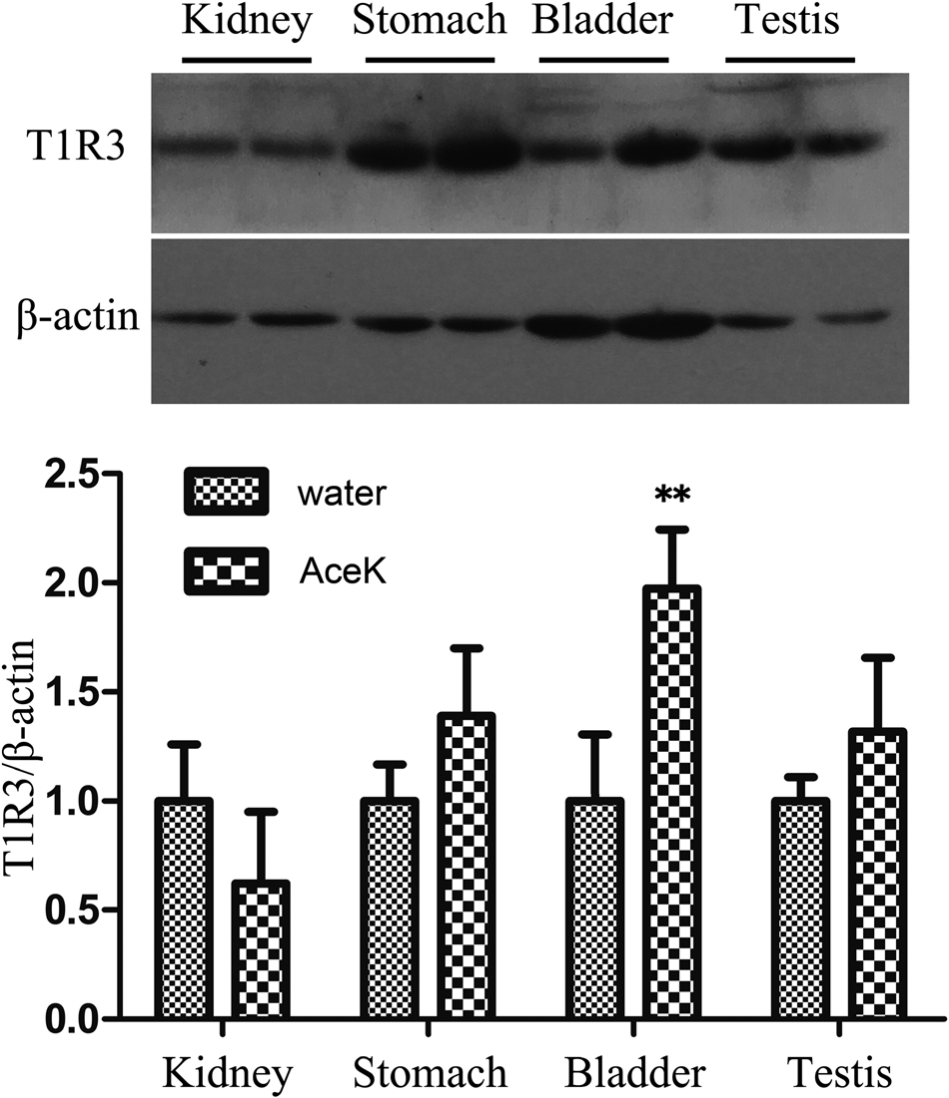
Expression levels of the sweet taste receptor T1R3 in the kidney, stomach, bladder, and testis by western blotting analysis. The target bands of sweet taste receptors were approximate 90 kDa. *n* = 10 in each group. AK acesulfame potassium. The water group was used as the negative control. The uncropped bolt image is shown in Supplementary Fig. S1. Student’s *t* test was performed for all results. Error bars show the standard deviations of the means. *P < 0.05; **P < 0.01.

Several relavant tissues, including the kidneys, stomach, bladder, and testes were investigated to evaluate the effect of high dose (10 mM) AK exposure, and the expression of sweet taste receptors was analyzed by western blotting. It was shown that one band migrating at a molecular weight of approximately 90 kDa were obtained, which was in agreement with theoretical molecular mass values calculated from the protein sequence. During the 4-week exposure, we found that the expression level of T1R3 in the bladder of the AK group was two-fold higher than that of the control group (*P* < 0.0001). Moreover, the T1R3 expression in the kidney was slightly down-regulated, whereas the protein levels of T1R3 in the stomach and in the testes were slightly increased, but were not statistically significant (Fig. 3).

**Fig. 4:**
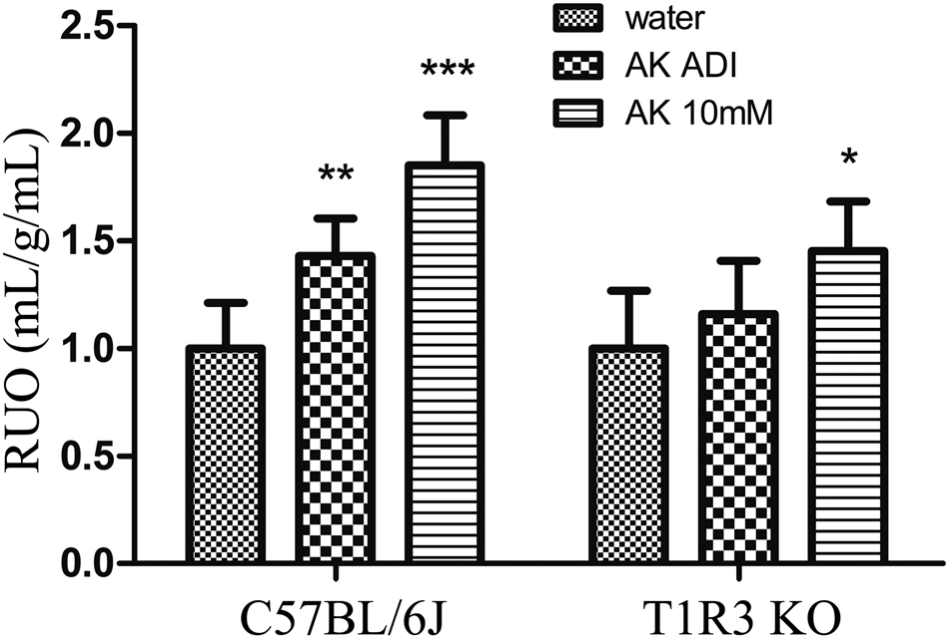
Relative urine output (RUO) in C57BL/6J and T1R3KO mice after orally exposure to AK for 4 weeks. *n* = 5 in each group. AK acesulfame potassium, The water group was used as the negative control. One-way ANOVA and Dunnett’s multiple comparison post-hoc test were performed for all results. Error bars show the standard deviations of the means. *P < 0.05; **P < 0.01; ***P < 0.001.

In order to verify whether sweet taste receptor contributed to the different urine volume. T1R3KO mice and C57BL/6J mice were used in the detection of urine output with the 4-week AceK administration. Similar changes of RUO were found in wild type C57BL/6J mice (Fig. 4). However, no significant differences were seen in the ADI group of T1R3KO mice. In addition, the RUO in 10 mM group was reduced relative to the same concentration group in C57BL/6J mice, but still significantly increased compared with controls in T1R3KO mice (Fig. 4).

### Responses of sweet taste receptors T1R2/T1R3 in bladder to different artificial sweeteners

**Fig. 5:**
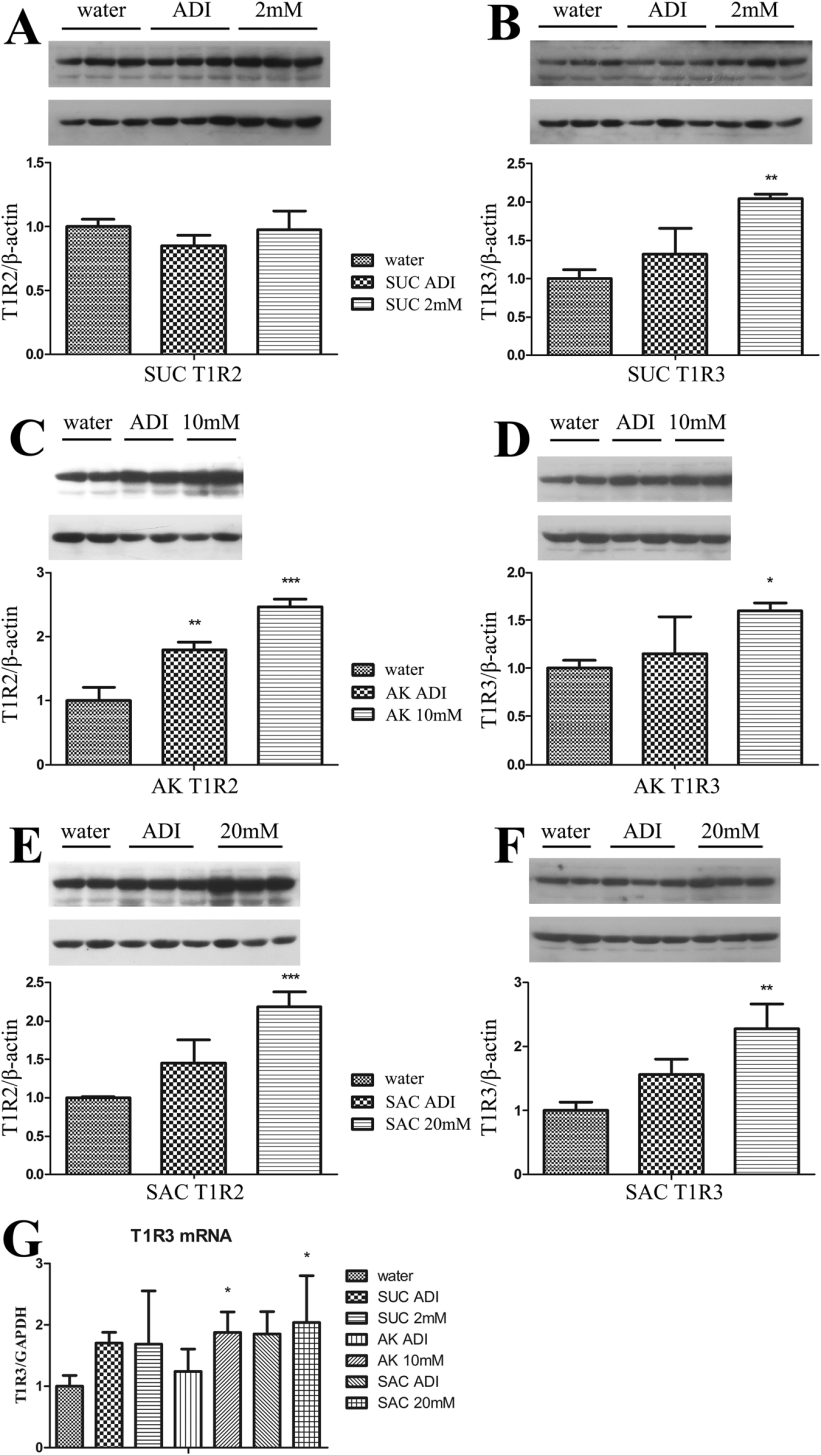
Increased T1R2/T1R3 expression in response to sweeteners given at low or high doses for 4 weeks to mice (*n* = 10). Sweet taste receptors T1R2 and T1R3 (approximate 90 kDa in all the graphs) expression levels from isolated bladder tissue of different groups were detected by western blots. Protein (50 μg) was loaded for each sample and separated by SDS-PAGE, using *β*-actin as an internal loading control. **A**, **B** were T1R2 and T1R3 protein expression levels for SUC (sucralose) group (low and high dose), respectively. **C**, **D** were T1R2 and T1R3 protein expression levels for AceK (acesulfame potassium) group (low and high dose), respectively. **E**, **F** were T1R2 and T1R3 protein expression levels for saccharin (saccharin) group (low and high dose), respectively. **G** mRNA levels of *tas1r3* were determined by qPCR and was standardized to GAPDH mRNA. Results for each show relative *tas1r3* mRNA expression compared with the water (control) group. SUC sucralose, AK acesulfame potassium, SAC saccharin. The water group was used as the negative control. Graph **B**, **D**, **F** shared the same legend with **A**, **C**, **E**, respectively. The uncropped bolt images are shown in Supplementary Figs. S2–S10. One-way ANOVA and Dunnett’s multiple comparison post-hoc test were performed for all results. All the error bars show the standard deviations of the means. *P < 0.05; **P < 0.01; ***P < 0.001.

To determine whether the changes in urinary excretion corresponded to the changes in sweet receptor expression of bladder, we examined the expression of sweet-sensing genes *tas1r2/tas1r3* at both the mRNA and protein levels by qPCR and western blot analyses, respectively (Fig. 5). At the protein level, T1R3 expression in the high dose groups for all the sweeteners was significantly higher than that in the control group. Although the T1R3 protein expression was slightly increased in the low-dose groups as well, it was not statistically significant (Fig. 5B, D, F). Every dose of the different sweeteners increased the T1R3 protein expression relative to the control group, and a gradually increasing trend from the low-dose to the high-dose of sweetener was found for all the ASs. On the other hand, the profile of T1R2 expression was somewhat different. In the sucralose group, there was no significant change at either sweetener concentrations (Fig. 5A). In the AceK-treated group, the T1R2 expression was significantly enhanced at both doses. Moreover, T1R2 expression in the high-dose AceK-treated group was over two-fold higher than that of the control group (Fig. 5C). A similar increase was observed in the high-dose saccharin group, but no obvious change was found in the low-dose group (Fig. 5E). In the qPCR analysis, several specific primer pairs designed for *tas1r2* were tested, unfortunately, none of which were able to evaluate the gene expression in a credible range (the CT value was more than 37–38 cycles). However, the gene expression of *tas1r3* was detectable with the primer pairs used (CT value was less than 30 cycles). The results showed that the *tas1r3* mRNA expression level was significantly increased at the high-dose of saccharin-treated and AceK-treated groups relative to the control group. The saccharin-treated group had over a two-fold increase compared to the control group in its mRNA expression. The T1R3 expression level in both sucralose dose groups and the low-dose saccharin group were also increased, but they were not significant (Fig. 5G).

## Discussion

This study presents results from an experiment aimed at testing the effect of long-term exposure (4 weeks) to several ASs on urine output in mice. In order to reduce the difference in urination caused by the different water intake, we chose different concentration for different sweeteners to keep similar daily drinking volume of different sweeteners. The pre-experiment of drinking curve of different sweeteners in different concentration showed that, in the ADI based lower concentration sweetener group, the mice showed a preference of sweetener solution close to that of pure water, and in the higher concentration sweetener group, the mice showed significant preference for sweetener solution. Between the three sweeteners, the average daily drinking volume by the sucralose solution of sweetness 24, saccharin solution of sweetness 30 and acesulfame K solution of sweetness 20 were similar for the preferences of mice (data not shown).

After the administration, the saccharin and AceK were shown to increase urine output in C57BL/6J mice at higher concentrations and AceK also increased urine output at the lower concentration. However, sucralose did not appear to increase urine output at any concentration. As reported, the absorption of AceK and saccharin in gastrointestinal tract was much higher than that of sucralose^24^, suggesting that the effect of sucralose on bladder cannot be calculated according to the amount of drinking consumption, which may be the reason why no significant change was found in the experiment of urination.

In this study, the increasing urine output by AS administration was shown to be related to the sweet taste receptors by comparing the wild type C57BL/6J mice to T1R3 knock out mice. The successful quantitation of T1R2/T1R3 strongly supports our hypothesis that the ASs have an effect on the urine output owing to the altered expression of sweet taste receptors. The T1R3KO mice experiment demonstrated the correlation between sweet taste receptor and RUO (Fig. 4). In the ADI dose group of AceK, the RUO of T1R3KO mice were not as significantly different as in wild type (WT) C57BL/6J mice. In addition, there was also a weakening trend of RUO in the high dose group in T1R3KO mice compared with WT C57BL/6J mice. This indicates that AceK may exert its effects on RUO through multiple, independent mechanisms. One mechanism could be mediated by sweetness receptors, as supported by changes in T1R3 expression across various tissues. Another mechanism could be directly associated with alterations in urine production capabilities, which would implicate renal function. As we all knew, the kidney is the most closely related organ for urine output. Combined with the T1R3 expression levels in multi-tissues after AS exposure, it is interested to note that no significant difference of T1R3 expression was detected in the kidney. T1R3 expression levels were up-regulated after the AS exposure only in the bladder among all the screened tissues (Fig. 3). Further detection on the expression levels of T1R2/T1R3 in the bladder exposed to different concentrations of three ASs, we found that all the ASs with elevated urination significantly up-regulated the T1R2/T1R3 in the bladder of mice (Fig. 5). Previous study has shown that overactive bladder can lead to frequent urination and urgency, which may increase urine volume^18^. Another study indicated that saccharin intake might lead to overactive bladder^17^. This may suggest the physiological basis of the increase in urine output induced by ASs intake.

As reported, the volume of urine excretion is mainly determined by ion intake and water intake^25^. In this study, there is no significant difference in ion intake among all mice. The results showed that the intake of AceK had the greatest effect on RUO, and saccharin could significantly increase RUO only at higher concentrations (Fig. 1D). These may result in the weight loss in high concentration of AceK group and saccharin group (Fig. 1A). Although some researchers had shown that the use of ASs could lead to weight gain^26^, there were no consistent conclusions in this field^27^, which might be related to the different feeding conditions and concentrations of ASs.

The concentration of ions or electrolytes we tested in urine is changed by most sweeteners used in this study, especially in the high dose group. It seems to be that, in addition to renal reabsorption of different ions that may be affected by sweeteners, changes in bladder contraction caused by AS intake may also be responsible^17,23^. Low-calorie ASs can activate the signaling cascade associated with T1R2/T1R3 receptors and gustducin protein Gα to trigger calcium-mediated signaling pathways^9^, which are vital in many physiological processes including the ion balance in body fluid. In the analysis of ion concentration in urine, the variation trend of K^+^ concentration had a better correlation with the change of RUO (Figs. 1D and 2). We speculated that K^+^ might be a direct or indirect factor of the changes in urinary excretion after ASs exposure. At present, no study has reported that K^+^ was directly related to urinary output, in neither kidney nor bladder. In this study, the concentrations of Na^+^ were not significantly change; this is because the intake of Na^+^ in our study was much lower than that of in previous experiments^28^. It was worth mentioning that the saccharin group decreased significantly in the detection of Na^+^ and Cl^-^ (Fig. 2), and the reason is still unknown. The changes of Cl^-^ may be synergistic with Na^+^, and there seems to be no significant difference in Ca^2+^.

In order to truly find the most related pathway, a tissue-specific T1R3 knockout mouse would have to be constructed in future study. Though the expression level of sweet taste receptors of bladder was indicated to be positively related to the urine output in our study, the increased urine output could also be due to other tissues, like stomach (expression in the stomach is shown in Fig. 3) or gut communicating with other organs like the bladder through hormonal or other signals. T1R2/3 in the gut enteroedocine cells and/or epithelium has been suggested to signal to pancreatic beta cells and other gut epithelial cells through secretion of GLP-1/2 peptides. A systemic pathological analysis of kidney of sweetener exposed mice was needed to further study the relationship between the altered ion concentration of urine and the up-regulated expression of bladder sweet taste receptors.

## Methods

### Animals housing and samples preparation

All the experiments performed on animals were consistent with the regulations for Laboratory Animal Welfare and Ethics Committee. The protocols were approved by the Institutional Animal Care and Use Committee of Zhejiang Gongshang University (No. 2018R06). The C57BL/6J male mice were purchased from Shanghai Slack Experimental Animal Limited Liability Company. The *tas1r3* knockout mice (T1R3KO) were obtained from Monell Chemical Senses Center. Animals were individually housed in a controlled environment, the feeding room’s temperature was about 25 °C, and the humidity was approximately 40%. The room was automatically controlled at a 12 h light/12 h dark cycle, with the light period beginning at 8:00 a.m. All groups were fed the normal rodent diet (GB 14924-2001, China). Experiments took place after a one-week acclimatization period. In the first assay, a total 70 individually housed C57BL/6J male mice were divided into seven groups, with 10 mice per group, and exposed to water or solutions of sweeteners with different sweeteners in an ADI or higher dose for 4 weeks, respectively. Then another 15 individually housed C57BL/6J male mice and 15 T1R3KO mice were divided into six groups, with 5 mice per group, and exposed to water or solutions of sweeteners with AceK in an ADI or higher dose, respectively. All ASs were purchased from Aladdin. Body weight and drinking consumption were measured every two or three days. Daily drinking was calculated as the consumption volume per gram of body weight in this study. Mice were euthanized by cervical dislocation. The tissues, including kidneys, stomach, bladder, and testes, were immediately excised and rinsed with ice-cold physiological saline. Each bladder tissue was split into two parts, immersed in liquid nitrogen, and stored at −80 °C for further use.

### Preparation of the aqueous solutions of sweeteners

All the solutions of sucralose, acesulfame K or saccharin were prepared in deionized water. In this study, the intensity of sweetness was converted relative to 2% sucrose as sweetness unit 1^29^. The relative sweetness of sucralose, acesulfame K or saccharin were 600, 200 and 160, respectively^30^. The designed concentration of each solution was calculated as listed in Table 1. The final concentrations of aqueous solutions of sweeteners (C) were calculated as Eq. (1):

1 $${\rm{C}}=({\rm{N}}* {\rm{Sd}})/({\rm{Sc}}* {\rm{Mw}})$$

**Table 1:** The calculation and concentrations of solutions of sweeteners used in this study.

Group	ADI (mg Kg^−1^ body weight)	Molecular Weight (MW)	Relative sweetness to sucrose (Sc)	Designed sweetness in pre-test	Amount of daily drinking (mL)	Calculated concentration (mM)	Designed concentration (C, mM)	Designed sweetness (Sd)
Low Sucralose (ADI)	15	397.6	600	-	5.0–6.0	0.16–0.19	0.18	2.1
High Sucralose				24.0	8.0–10.0	-	2	24
Low Saccharin (ADI)	5	183.2	160	-	5.0–6.0	0.11–0.14	0.12	0.7
High Saccharin				30.0	8.0–10.0	-	20	30
Low AK (ADI)	9	201.2	200	-	5.0–6.0	0.19–0.22	0.20	1.2
High AK				20.0	8.0–10.0	-	10	20

*N* = 20 (gram/L, 2% sucrose).

*C* designed concentration of sweetener solution (mol / L), *Sd* designed sweetness (preference for sweetener solution of mice),

*Sc* relative sweetness of sweetener to sucrose,

*Mw* molecular weight of sweet molecule.

In briefly, the sweetness of sucralose higher dose group was firstly set to 24, the similar drinking consumption of high dose acesulfame K and saccharin was closely to 20 and 30, respectively. The data of daily drinking of solutions with different sweetness were obtained from our unpublished data and precious study^31^. The concentration of three ADI groups were calculated based on the amount of the ADI of each sweetener and daily drinking mentioned above (approximate weight of an adult C57BL/6J mouse was set to 30 g). The drinking consumption of the three ADI groups seem to not be significant different.

### Detection of urine output and ion concentration

Before urine collection, all groups were supplied with sufficient water to replace the AS solutions for 48 h. Then, a 24-h urine output was measured in an ecological cage (BW-FMS501, Shanghai, China). After 24 h, the urine was collected, the volumes were measured, and the water intake and the body weight of each mouse were recorded. In order to reduce urine volatilization and microbial contamination, we conducted ice bath on the urine collector, and renewed the ice bath module every 8 h. The RUO (comparison between mice of the same genotype) was calculated as Eq. (2):

2 $${\rm{RUO}}=\,\frac{\left({\rm{urine}}\; {\rm{volume}}\right){\rm{mL}}}{\left({\rm{water}}\; {\rm{intake}}\right){\rm{mL}}\,*\, \left({\rm{body}}\; {\rm{weight}}\right){\rm{g}}}$$

The concentration of Na^+^, K^+^, Cl^-^ and Ca^2+^ were measured using Synchron CX9 biochemistry analyzer (Beckman Coulter, USA) following manufacturer’s instructions.

### Real-Time Quantitative PCR (RT-qPCR)

Total RNA was isolated from the frozen urinary bladder, using Trizol (15596018, Thermo Fisher, USA). The RNA was quantified using a Nanodrop 2000 (Thermo Fisher, USA). The RNA was transcribed into cDNA, using HiScript Q RT SuperMix for qPCR (R123-01, Vazyme, China). Quantitative PCR was conducted in 20 μL reactions using AceQ qPCR SYBR Green Master Mix (Q111-02/03, Vazyme, China). The following PCR primers for mouse T1Rsand GAPDH were purchased from Invitrogen: for mouse T1R3, 5’-CAGTCAAAGCATTGCTGCCT-3’ (forward) and 5’-ATAGCTGACCTGTGGCATGA-3’ (reverse); for mouse T1R2-1, 5’-TGGCAGCTATGGTGACTACG-3’ (forward) and 5’-CAGCACCACAGACCTGAAGA-3’ (reverse); for mouse T1R2-2, 5’-GCACCAAGCAAATCGTCTATCC-3’ (forward) and 5’-ATTGCTAATGTAGGTCAGCCTCGTC-3’ (reverse); for mouse GAPDH,5’-CAGGACGCATTGCTGACAAT-3’ (forward) and 5’-TCTTCACCACCATGGAGAAG-3’ (reverse).

The relative gene expression ratios between different groups were calculated according to a comparative Ct method (ΔΔCt value) with StepOne Real-Time PCR System (4376374, Thermo Fisher, USA). The reaction system was incubated at 95 °C for 7 min. A three-step PCR procedure was followed: 15 s at 95 °C, 20 s at 60 °C, then 20 s at 72 °C for 40 cycles. GAPDH was used as an internal reference.

### Western blot assay

Following the protocol reported before^32^, the abundance of T1R2/T1R3 protein was assessed by western blot relative to the endogenous *β*-actin expression as a loading control. The specificity of primary antibodies against T1R2 and T1R3 were previous tested^33^. In briefly, the same quantity of protein sample (50 μg) from each experiment was loaded and separated by polyacrylamide gel electrophoresis on a 10% SDS gel, and then transferred electronically to a PVDF membrane (1620177, Bio-Rad, USA). The PVDF membrane was blocked in TBS/5% skim milk at 4 °C overnight. The following day, the membrane was incubated with a primary antibody either against T1R2 (sc-50306, Santa Cruz, USA, 1:1000), T1R3 (sc-50352, Santa Cruz, USA, 1:1000), or *β*-actin (R1207, Huaan Biotec, China, 1:2000) at 4 °C for 10 h, followed by incubation with goat anti-rabbit IgG antibodies (GAR007, Multi Sciences, China, 1: 2000) for 1 h at room temperature. The PVDF membrane was treated with BeyoECL Star solution (P0018A, Beyotime, China) and then exposed on an X-ray film (Carestream, Canada) in a darkroom. All blots derive from the same experiment and that they were processed in parallel. Images were scanned to perform densitometry analysis using Image J, using *β*-actin as the internal loading control. The uncropped bolt images are shown in supplementary data.

### Statistical analysis

The results are expressed as means ± SD. All data of western blotting were analyzed using GraphPad Prism 5.0 software (San Diego, USA). One-way ANOVA, Dunnett’s multiple comparison post-hoc test, or Student’s *t* test was performed for the results. Three independent experiments were performed for each result.

### Reporting summary

Further information on research design is available in the Nature Research Reporting Summary linked to this article.

### Supplementary information

SUPPLEMENTARY DATA

reporting summary

## Data Availability

The authors declare that all data supporting the findings of this study are available in the paper.

## References

[1] Fernstrom JD (2015). Non-nutritive sweeteners and obesity. Annu. Rev. Food Sci. Technol..

[2] Shi Q (2019). Low intake of digestible carbohydrates ameliorates duodenal absorption of carbohydrates in mice with glucose metabolism disorders induced by artificial sweeteners. J. Sci. Food Agric..

[3] Bleich SN, Wolfson JA, Vine S, Wang YC (2014). Diet-beverage consumption and caloric intake among US adults, overall and by body weight. Am. J. Public Health.

[4] Nettleton JA (2009). Diet soda intake and risk of incident metabolic syndrome and type 2 diabetes in the Multi-Ethnic Study of Atherosclerosis (MESA). Diabetes Care.

[5] Tandel KR (2011). Sugar substitutes: health controversy over perceived benefits. J. Pharm. Pharmacother..

[6] Chandrashekar J, Hoon MA, Ryba NJ, Zuker CS (2006). The receptors and cells for mammalian taste. Nature.

[7] Zhang Y (2003). Coding of sweet, bitter, and umami tastes: different receptor cells sharing similar signaling pathways. Cell.

[8] Tordoff MG, Alarcon LK, Valmeki S, Jiang P (2012). T1R3: a human calcium taste receptor. Sci. Rep..

[9] Zhao GQ (2003). The receptors for mammalian sweet and umami taste. Cell.

[10] Tordoff MG (2001). Calcium: taste, intake, and appetite. Physiol. Rev..

[11] Yoshida R, Ninomiya Y (2016). Taste information derived from T1R-expressing taste cells in mice. Biochem. J..

[12] Kiuchi S (2006). Genomic structure of swine taste receptor family 1 member 3, TAS1R3, and its expression in tissues. Cytogenet. Genome Res..

[13] Laffitte A, Neiers F, Briand L (2014). Functional roles of the sweet taste receptor in oral and extraoral tissues. Curr. Opin. Clin. Nutr. Metab. Care.

[14] Hass N, Schwarzenbacher K, Breer H (2010). T1R3 is expressed in brush cells and ghrelin-producing cells of murine stomach. Cell Tissue Res..

[15] Widmayer P (2012). Altered expression of gustatory-signaling elements in gastric tissue of morbidly obese patients. Int. J. Obes..

[16] Gong T, Wei Q, Mao D, Shi F (2016). Expression patterns of taste receptor type 1 subunit 3 and alpha-gustducin in the mouse testis during development. Acta Histochem..

[17] Bakali E, Hong J, Gillespie J, Tincello D (2017). Saccharin increases perception of bladder filling in a forced diuresis experiment. Neurourol. Urodyn..

[18] Gormley EA (2012). Diagnosis and treatment of overactive bladder (Non-Neurogenic) in adults: AUA/SUFU Guideline. J. Urol..

[19] Renwick AG (1985). The disposition of saccharin in animals and man-a review. Food Chem. Toxicol..

[20] Whitehouse CR, Boullata J, McCauley LA (2008). The potential toxicity of artificial sweeteners. Aaohn J..

[21] Whysner J, Williams GM (1996). Saccharin mechanistic data and risk assessment: urine composition, enhanced cell proliferation, and tumor promotion. Pharmacol. Ther..

[22] Elliott RA, Kapoor S, Tincello DG (2011). Expression and distribution of the sweet taste receptor isoforms T1R2 and T1R3 in human and rat bladders. J. Urol..

[23] Dasgupta J, Elliott RA, Doshani A, Tincello DG (2006). Enhancement of rat bladder contraction by artificial sweeteners via increased extracellular Ca2+ influx. Toxicol. Appl. Pharmacol..

[24] Magnuson BA (2016). Biological fate of low-calorie sweeteners. Nutr. Rev..

[25] Stanhewicz AE, Kenney WL (2015). Determinants of water and sodium intake and output. Nutr. Rev..

[26] Bian X (2017). The artificial sweetener acesulfame potassium affects the gut microbiome and body weight gain in CD-1 mice. PLoS One.

[27] Rogers PJ (2016). Does low-energy sweetener consumption affect energy intake and body weight? A systematic review, including meta-analyses, of the evidence from human and animal studies. Int J. Obes..

[28] Fenner-Crisp PA (1995). Urinary bladder carcinogenesis. Toxicol. Pathol..

[29] Hall J. E. & Guyton A. C. Guyton and Hall textbook of medical physiology. 12th ed. 645–647 (Saunders/Elsevier, 2011).

[30] Steinert RE (2011). Effects of carbohydrate sugars and artificial sweeteners on appetite and the secretion of gastrointestinal satiety peptides. Br. J. Nutr..

[31] Bachmanov AA, Tordoff MG, Beauchamp GK (2001). Sweetener preference of C57BL/6ByJ and 129P3/J mice. Chem. Senses.

[32] Shi Q, Zhu X, Zhou J, Chen L (2018). Low intake of digestible carbohydrates ameliorates the duodenal absorption of carbohydrates in mice with glucose metabolic disorders induced by sucralose. Food Funct..

[33] Harrington EO (2018). Activation of the sweet taste receptor, T1R3, by the artificial sweetener sucralose regulates the pulmonary endothelium. Am. J. Physiol. Lung Cell. Mol. Physiol..

