# Supplementary material for: Sweet taste receptors play roles in artificial sweetener-induced enhanced urine output in mice
Source: NPJ Sci Food. 2024 Jan 5;8:2. doi: 10.1038/s41538-023-00236-9 (PMC10770165; doi:10.1038/s41538-023-00236-9)

ALL THE UNCROPPED BLOT IMAGES

Figure S1. The uncropped bolt image of figure 3


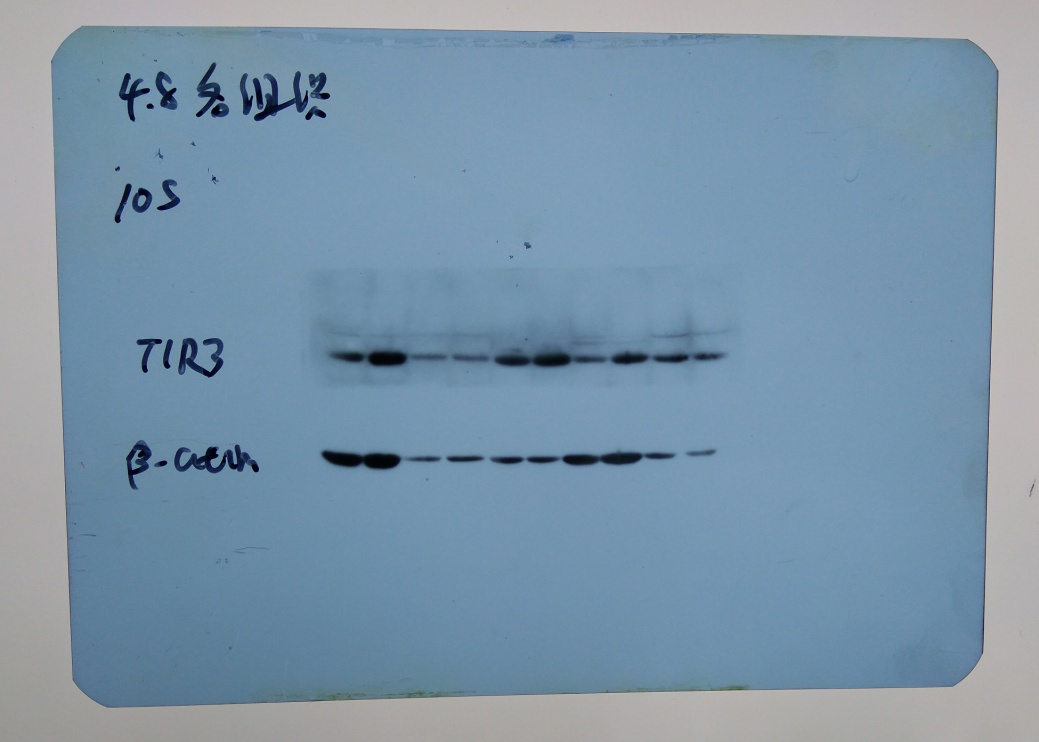


Figure S2. The uncropped bolt image of figure 5A


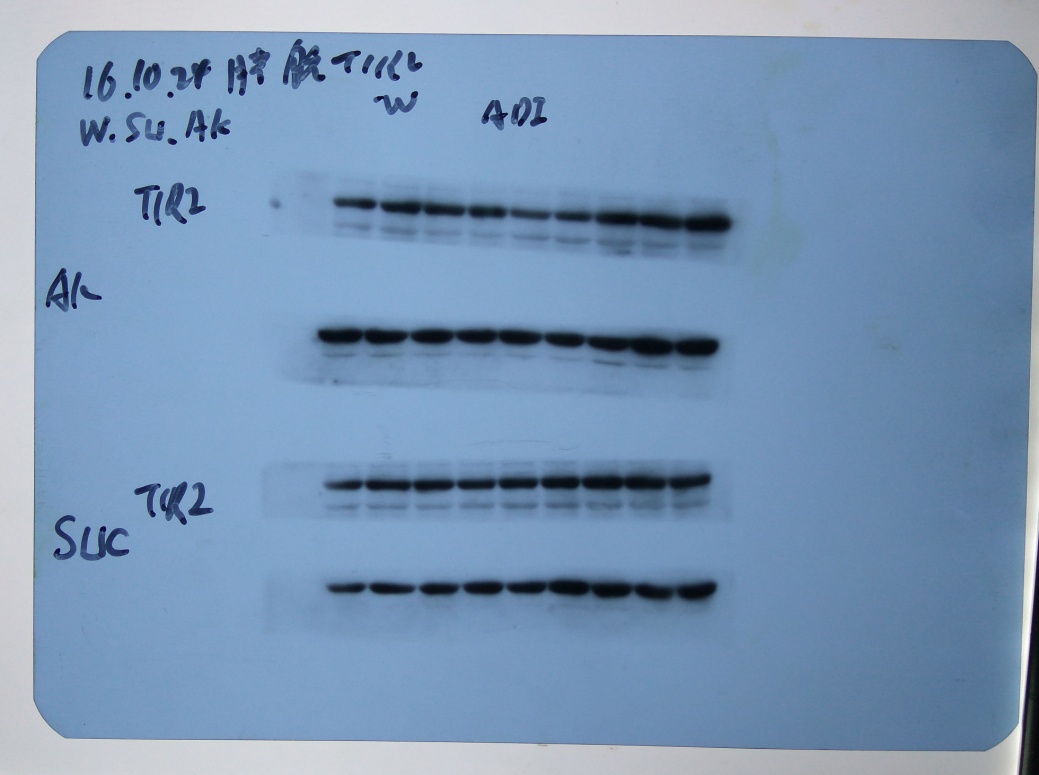


Figure S3. The uncropped bolt image of figure 5B (anti T1R3)


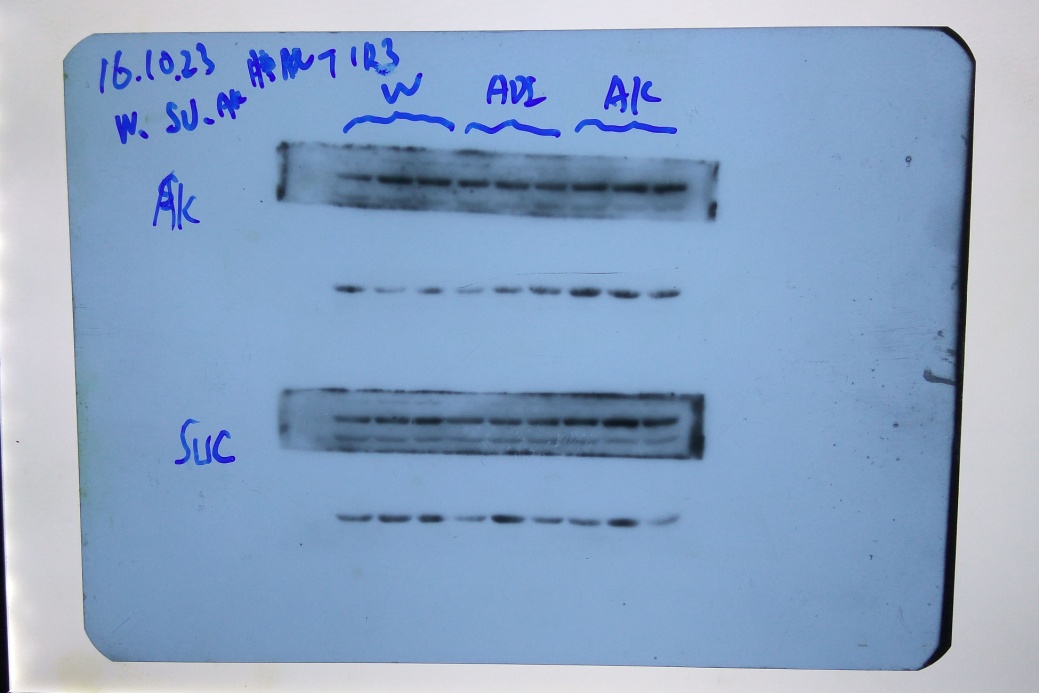


Figure S4. The uncropped bolt image of figure 5B (anti actin)


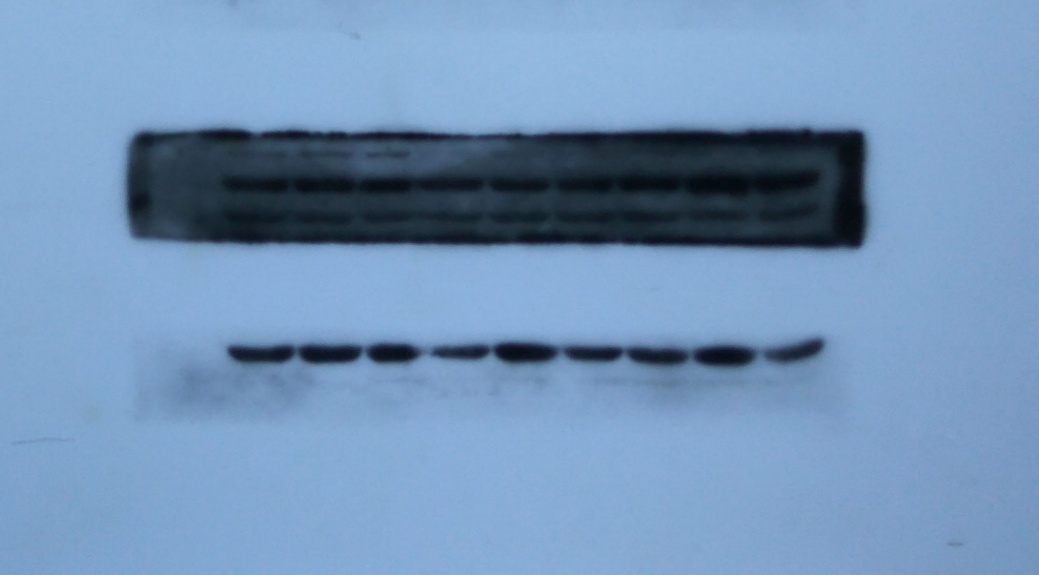


Figure S5. The uncropped bolt image of figure 5C


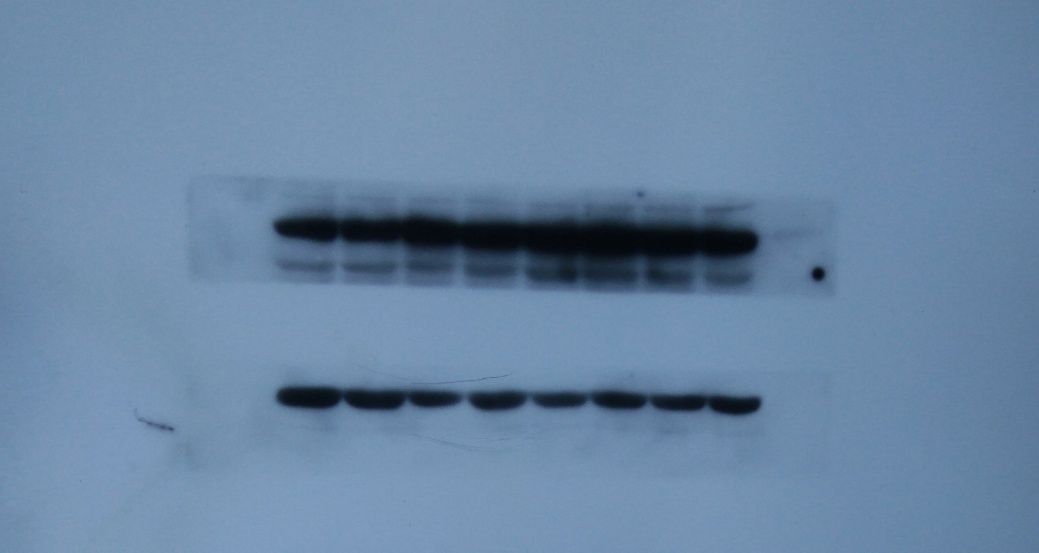


Figure S6. The uncropped bolt image of figure 5D (anti T1R3)


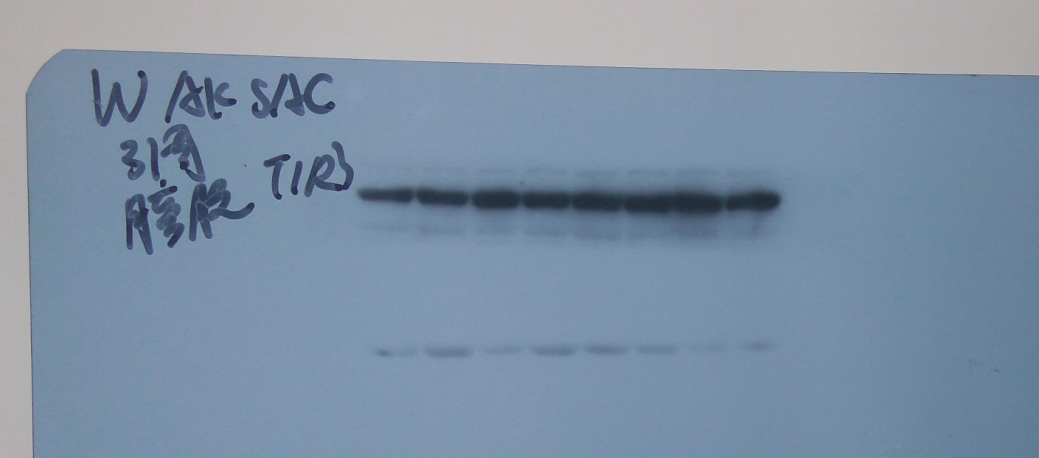


Figure S7. The uncropped bolt image of figure 5D (anti actin)


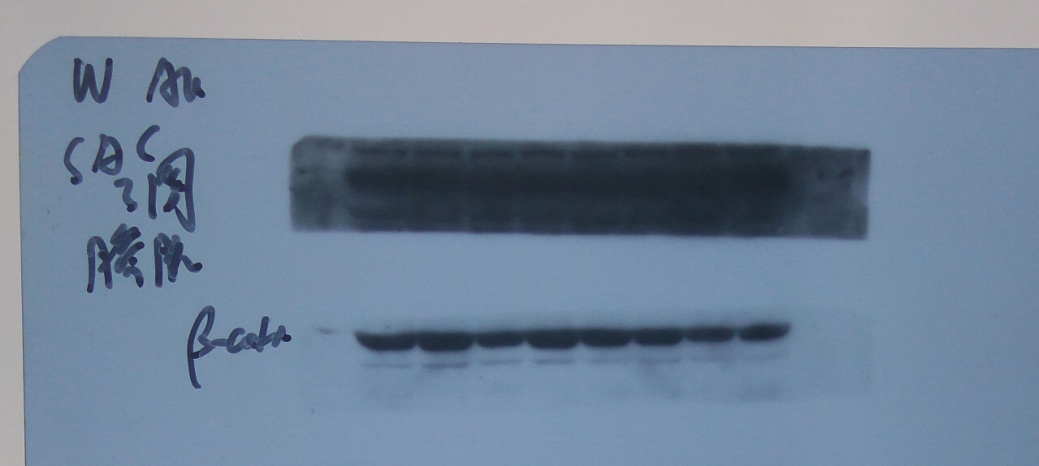


Figure S8. The uncropped bolt image of figure 5E


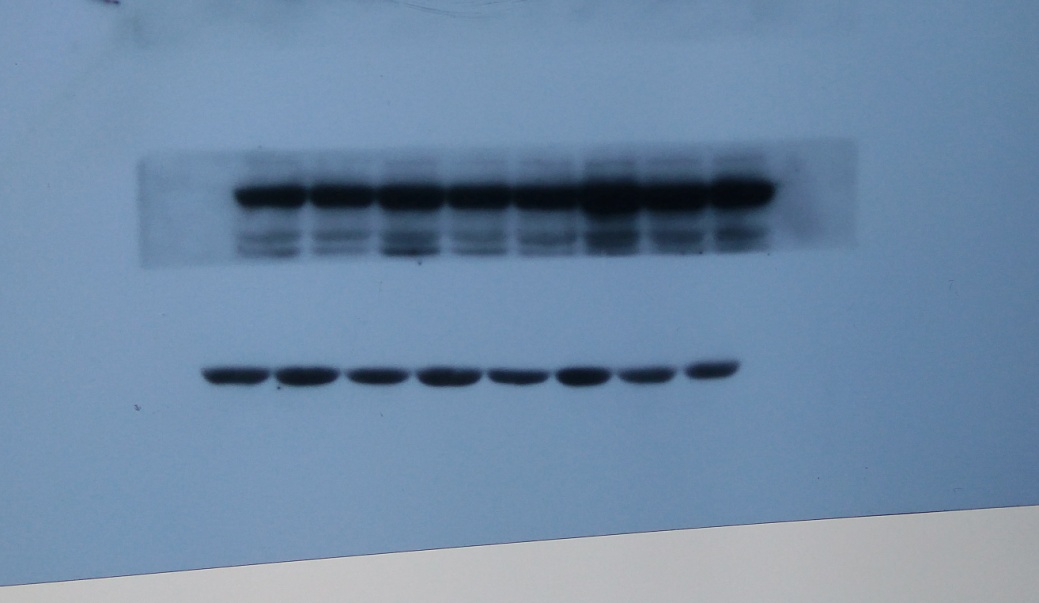


Figure S9. The uncropped bolt image of figure 5F (anti T1R3)


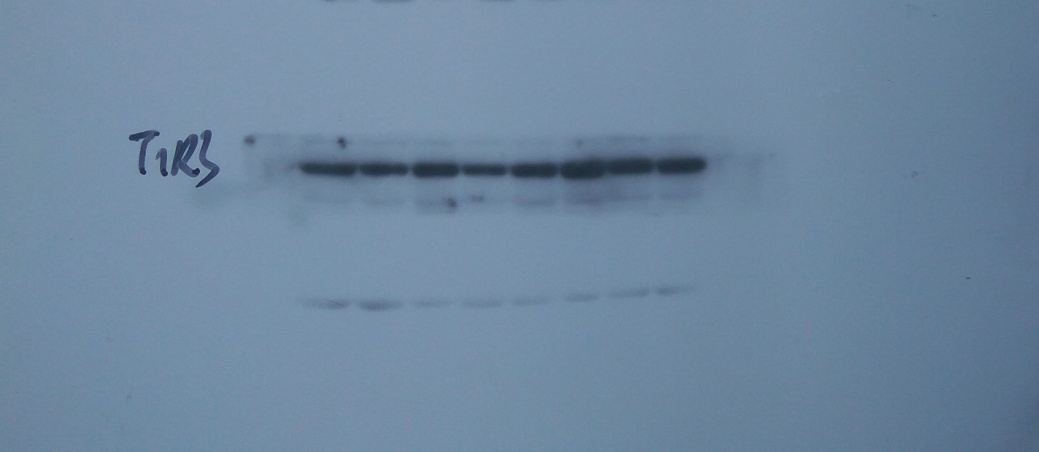


Figure S10. The uncropped bolt image of figure 5F (anti actin)


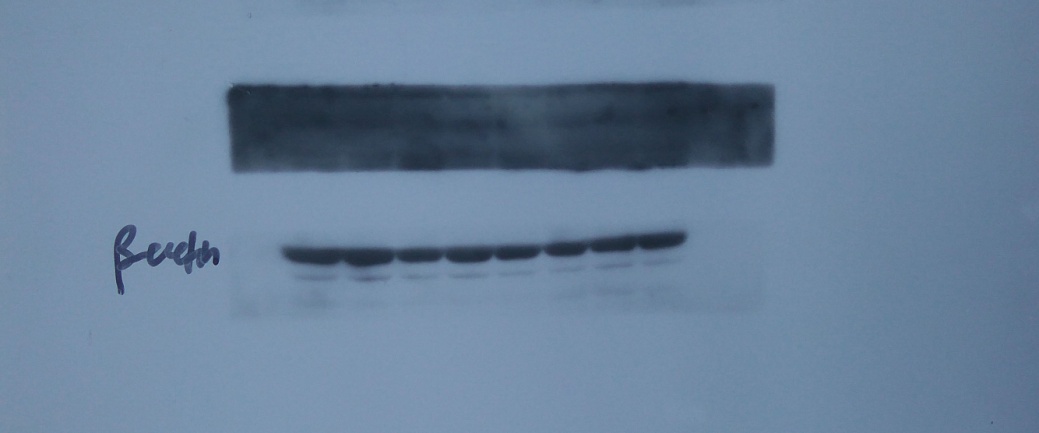

Supplement: Supplementary file 1 — SUPPLEMENTARY DATA [file 41538_2023_236_MOESM1_ESM.docx]
