# Supplementary material for: Sweet taste receptors play roles in artificial sweetener-induced enhanced urine output in mice
Source: NPJ Sci Food. 2024 Jan 5;8:2. doi: 10.1038/s41538-023-00236-9 (PMC10770165; doi:10.1038/s41538-023-00236-9)
Supplement: Supplementary file 2 — reporting summary [file 41538_2023_236_MOESM2_ESM.pdf]

nature portfolio

Corresponding author(s): Lei Cai, NPJSCIFOOD-00945R2

Last updated by author(s): Oct 17, 2023

Reporting Summary

Nature Portfolio wishes to improve the reproducibility of the work that we publish. This form provides structure for consistency and transparency in reporting. For further information on Nature Portfolio policies, see our Editorial Policies and the Editorial Policy Checklist.

Statistics

For all statistical analyses, confirm that the following items are present in the figure legend, table legend, main text, or Methods section.

n/a	Confirmed	
☐	☒	The exact sample size (*n*) for each experimental group/condition, given as a discrete number and unit of measurement
☐	☒	A statement on whether measurements were taken from distinct samples or whether the same sample was measured repeatedly
☐	☒	The statistical test(s) used AND whether they are one- or two-sided<br>*Only common tests should be described solely by name; describe more complex techniques in the Methods section.*
☒	☐	A description of all covariates tested
☐	☒	A description of any assumptions or corrections, such as tests of normality and adjustment for multiple comparisons
☒	☐	A full description of the statistical parameters including central tendency (e.g. means) or other basic estimates (e.g. regression coefficient) AND variation (e.g. standard deviation) or associated estimates of uncertainty (e.g. confidence intervals)
☒	☐	For null hypothesis testing, the test statistic (e.g. *F*, *t*, *r*) with confidence intervals, effect sizes, degrees of freedom and *P* value noted<br>*Give P values as exact values whenever suitable.*
☒	☐	For Bayesian analysis, information on the choice of priors and Markov chain Monte Carlo settings
☒	☐	For hierarchical and complex designs, identification of the appropriate level for tests and full reporting of outcomes
☒	☐	Estimates of effect sizes (e.g. Cohen's *d*, Pearson's *r*), indicating how they were calculated

Our web collection on statistics for biologists contains articles on many of the points above.

Software and code

Policy information about availability of computer code

Data collection	No software was used.
Data analysis	Image J and GraphPad Prism 5.0

For manuscripts utilizing custom algorithms or software that are central to the research but not yet described in published literature, software must be made available to editors and reviewers. We strongly encourage code deposition in a community repository (e.g. GitHub). See the Nature Portfolio guidelines for submitting code & software for further information.

Data

Policy information about availability of data

All manuscripts must include a data availability statement. This statement should provide the following information, where applicable:

- Accession codes, unique identifiers, or web links for publicly available datasets
- A description of any restrictions on data availability
- For clinical datasets or third party data, please ensure that the statement adheres to our policy

The authors declare that all data supporting the findings of this study are available in the paper

Research involving human participants, their data, or biological material

Policy information about studies with human participants or human data. See also policy information about sex, gender (identity/presentation), and sexual orientation and race, ethnicity and racism.

Reporting on sex and gender	n/a
Reporting on race, ethnicity, or other socially relevant groupings	n/a
Population characteristics	n/a
Recruitment	n/a
Ethics oversight	n/a

Note that full information on the approval of the study protocol must also be provided in the manuscript.

Field-specific reporting

Please select the one below that is the best fit for your research. If you are not sure, read the appropriate sections before making your selection.

☒ Life sciences ☐ Behavioural & social sciences ☐ Ecological, evolutionary & environmental sciences

For a reference copy of the document with all sections, see nature.com/documents/nr-reporting-summary-flat.pdf

Life sciences study design

All studies must disclose on these points even when the disclosure is negative.

Sample size	For mouse experiments, there are usually around 5-10 mice in each group. In our study, we set up 10 mice in each group for behavioral research on wild type mice, and 5 mice in each group for gene knockout mice.
Data exclusions	no data were excluded from analyze.
Replication	Three independent experiments were performed for each result.
Randomization	In order to ensure the randomness of each group of mice in the experiment, we will mix and raise the required number of mice at the beginning of the experiment, plus an additional group of mice. After a week of adaptive cultivation, we will group and randomly grab 5 or 10 mice into one group, and raise them separately or in combination as needed.
Blinding	All experimental data were designed with a double blind design, and each independent experiment was completed by at least two individuals. Different individuals labeled the groups or samples with numbers that did not display relevant information.

Reporting for specific materials, systems and methods

We require information from authors about some types of materials, experimental systems and methods used in many studies. Here, indicate whether each material, system or method listed is relevant to your study. If you are not sure if a list item applies to your research, read the appropriate section before selecting a response.

Materials & experimental systems

n/a	Involved in the study	
☐	☒	Antibodies
☒	☐	Eukaryotic cell lines
☒	☐	Palaeontology and archaeology
☐	☒	Animals and other organisms
☒	☐	Clinical data
☒	☐	Dual use research of concern
☒	☐	Plants

Methods

n/a	Involved in the study	
☒	☐	ChIP-seq
☒	☐	Flow cytometry
☒	☐	MRI-based neuroimaging

Antibodies

Antibodies used	primary antibody against T1R2 (sc-50306, Santa Cruz, USA), T1R3 (sc-50352, Santa Cruz, USA), or β-actin?R1207?Huaan Biotec, China?
Validation	The primary antibody against T1R2 and T1R3 used in this study were also used in published papers.<br>1. Harrington, E. O., Vang, A., Braza, J., Shil, A., & Chichger, H. (2018). Activation of the sweet taste receptor, t1r3, by the artificial sweetener sucralose regulates the pulmonary endothelium. American Journal of Physiology (1 Pt.1), 314.<br>2. Lizunkova, P., Enuwosa, E., & Chichger, H. (2018). Activation of the sweet taste receptor t1r3 by sucralose attenuates vegf-induced vasculogenesis in a cell model of the retinal microvascular endothelium. Graefes Archive for Clinical & Experimental Ophthalmology.<br>3. Shil, A.; Olusanya, O.; Ghufoor, Z.; Forson, B.; Marks, J.; Chichger, H. (2020) Artificial Sweeteners Disrupt Tight Junctions and Barrier Function in the Intestinal Epithelium through Activation of the Sweet Taste Receptor, T1R3. Nutrients, 12, 1862.<br><br>The primary antibody against β-actin used in this study was also used in published papers.<br>1. Wang C, Wei S, Liu B, Wang F, Lu Z, Jin M, Wang Y. Maternal consumption of a fermented diet protects offspring against intestinal inflammation by regulating the gut microbiota. Gut Microbes. 2022 Jan-Dec;14(1):2057779. doi: 10.1080/19490976.2022.2057779.<br>2. Sun F, Zhao Z, Willoughby MM, Shen S, Zhou Y, Shao Y, Kang J, Chen Y, Chen M, Yuan X, Hamza I, Reddi AR, Chen C. HRG-9 homologues regulate haem trafficking from haem-enriched compartments. Nature. 2022 Oct;610(7933):768-774. doi: 10.1038/s41586-022-05347-z.

Animals and other research organisms

Policy information about studies involving animals; ARRIVE guidelines recommended for reporting animal research, and Sex and Gender in Research

Laboratory animals	C57BL/6J and C57BL/6J T1R3 Knock Out mouse.
Wild animals	not involve.
Reporting on sex	Only C57BL/6J male mice were used in this study. In long-term dietary exposure experiments, female animals are prone to significant fluctuations in hormone levels, which can lead to significant standard deviations, often requiring more animals per group.
Field-collected samples	not involve.
Ethics oversight	All the experiments performed on animals were consistent with the regulations for Laboratory Animal Welfare and Ethics Committee. The protocols were approved by the Institutional Animal Care and Use Committee of Zhejiang Gongshang University (No. 2018R06).

Note that full information on the approval of the study protocol must also be provided in the manuscript.
